# Alpha-tocopherol serum concentrations and its relationship with anthropometric, biochemical, dietary and cardiovascular risk parameters

**DOI:** 10.3389/fnut.2025.1659286

**Published:** 2025-10-10

**Authors:** Ana Carolina Costa Campos Mota, Maria Clara da Cruz Carvalho, Mariana Duarte Bona, Daniele de Souza Marinho do Nascimento, Ingrid Naihara França de Sousa, Priscila Gomes de Oliveira, Eva Débora de Oliveira Andrade, Karla Danielly da Silva Ribeiro, Bruna Leal Lima Maciel

**Affiliations:** ^1^Graduate Program in Nutrition, Center for Health Sciences, Federal University of Rio Grande do Norte, Natal, Brazil; ^2^Graduate Program in Health Sciences, Center for Health Sciences, Federal University of Rio Grande do Norte, Natal, Brazil; ^3^Department of Medicine, Institute of Biomedicine, Federal University of Ceará, Fortaleza, Brazil; ^4^Department of Nutrition, Center for Health Sciences, Federal University of Rio Grande do Norte, Natal, Brazil

**Keywords:** diet, vitamin E, obesity, inflammation, heart disease risk factors

## Abstract

**Background:**

Alpha-tocopherol is a fat-soluble vitamin with antioxidant properties, capable of reducing oxidative stress and protecting cell membranes from oxidative damage. This vitamin also acts in the prevention of cardiovascular diseases, however research into this relationship is currently limited. This study aimed to assess the relationship between alpha-tocopherol concentrations and anthropometric, biochemical, usual dietary intake, and cardiovascular risk parameters.

**Methods:**

A cross-sectional study was conducted to collect sociodemographic, anthropometric, biochemical parameters of 92 adult individuals. Usual dietary intake was estimated with two 24-h recalls (24hR), using the Multiple Source Method. Cardiovascular risk was calculated using the Framingham global risk score (GRS). Alpha-tocopherol was examined by high performance liquid chromatography (HPLC). Multiple linear regression was used to evaluate the relationship between anthropometric, biochemical, usual dietary intake, and cardiovascular risk variables associated with alpha-tocopherol/total cholesterol concentrations.

**Results:**

The studied population presented a mean alpha-tocopherol of 17.80 μmol/L. Total cholesterol, non-HDL-c and LDL-c were significantly higher in individuals with higher serum concentrations of alpha-tocopherol. High-sensitivity C-reactive protein (hs-CRP) was significantly lower in subjects with higher concentrations of alpha-tocopherol. The GRS percentage was 10% for the total population. The multiple linear regression model showed that GRS was positively associated (β = 0.328; 95% CI 0.015, 0.100; *p* = 0.009) and the conicity index negatively associated (β = −0.290; 95% CI −8.196, −0.728; *p* = 0.020) with alpha-tocopherol/total cholesterol.

**Conclusion:**

Alpha-tocopherol was positively associated with biochemical and cardiovascular risk parameters, suggesting metabolic alterations that are related to low-grade inflammation resulting from excess weight and increased cardiovascular risk.

## 1 Introduction

Vitamin E is a fat-soluble group of eight compounds found in nature, divided into four tocopherols and four tocotrienols called alpha, beta, gamma, and delta, and alpha-tocopherol is the main form available in human plasma (1, 2). Vitamin E, due to its high concentration among the insoluble vitamin groups, regulates the redox balance and is present throughout the body, including cell membranes and lipoproteins, presenting antioxidant properties (3, 4). Vitamin E is also recognized for its anti-proliferative, pro-apoptotic, anti-angiogenic and anti-inflammatory effects (1).

In the diet, the main food sources of vitamin E are polyunsaturated vegetable oils (soybean, sunflower, and corn), nuts, seeds, eggs, liver, green vegetables, and dairy products (5, 6). Studies have shown that the intake of vitamin E seems to bring beneficial effects for cardiovascular health, being an important micronutrient in the prevention of cardiovascular diseases (7). On the other hand, high intake of alpha-tocopherol may be associated with impaired blood clotting, increased risk of bleeding and cardiovascular disease (8). Regarding vitamin E deficiency, neurological dysfunctions, muscle deterioration and a compromised immune system were observed (6, 9). As for alpha-tocopherol serum concentrations, values lower than 12 μmol/L indicate deficiency, while values higher than or equal to 30 μmol/L have been associated with a possible protective effect against cardiovascular diseases (10, 11).

The availability of this micronutrient seems correlated with nutritional status, especially obesity (1, 12). The chronic low-grade inflammation induced by excess weight increases oxidative stress, demanding antioxidants like vitamin E, and may lead to greater use and, consequently, a reduction in serum concentrations (13). Despite this assumption, the relationship between obesity and alpha-tocopherol status has not yet been fully elucidated due to the different results published, as some studies have found increased serum concentrations (14, 15) of this vitamin and others decreased in obesity (16, 17).

Obesity is also a risk factor for cardiovascular diseases (CVD), which are the leading causes of death and disability among overweight or obesity (18). Risk factors for cardiovascular events and deaths include hypertension, dyslipidemia, diabetes, obesity, diet, physical inactivity, and smoking (19). These components are associated with increased oxidation of low-density lipoprotein (LDL) cholesterol and the release of inflammatory cytokines (7).

Vitamin E is one of the most promising micronutrients for preventing CVD because of its antioxidant and anti-inflammatory properties (20). Low alpha-tocopherol concentrations have been associated with an increased incidence of CVD, while higher concentrations of this vitamin offer cardiovascular protection (21, 22). However, the studies that have evaluated this relationship present divergent results, reinforcing the need for further research (23, 24).

Thus, the aim of this study was to evaluate the relationship between alpha-tocopherol concentrations and anthropometric, biochemical, usual dietary intake, and cardiovascular risk indicators. Considering the evidence, the hypothesis under study is that lower serum concentrations of alpha-tocopherol are related to worse anthropometric, biochemical, usual dietary intake, and cardiovascular risk indicators.

## 2 Materials and methods

### 2.1 Ethical aspects

This study is part of a larger project, approved by the Research Ethics Committee (CEP) of the Onofre Lopes University Hospital of the Federal University of Rio Grande do Norte (HUOL/UFRN), CAAE n° 18923719.0.0000.5292, number 3.623.997. All eligible participants were informed of the study’s aims, risks, and benefits, and only those who agreed and signed the informed consent form participated. This study was conducted following all the ethical principles established in the Declaration of Helsinki (25).

### 2.2 Study design, participants and data collection

This is a cross-sectional study with data collection in two periods: October 2019 to March 2020 (first period interrupted due to the COVID-19 pandemic) and November 2021 to March 2023, in Natal, Rio Grande do Norte, Brazil. Convenience sampling was carried out, including volunteers over the age of 18 of both sexes, who agreed to participate in the study. Exclusion criteria were pregnant and/or breastfeeding women or individuals with cognitive impairment who were unable to answer the questionnaires, people using antimicrobials, antivirals or using vitamin E supplements, undergoing chemotherapy and/or radiotherapy or who had infection symptoms, such as diarrhea, vomiting or fever.

In total, 94 individuals were recruited to take part in the study through social media and/or telephone contact. Of these, 1 was excluded due to a low BMI and 1 due to the impossibility of collecting blood. In the end, 92 individuals took part in the study.

Sociodemographic data (age, gender, race, family income), anthropometric data (weight, height, waist, and hip circumference), lifestyle data (alcohol consumption, smoking, sedentary lifestyle), and self-reported diagnosis of chronic non-communicable diseases were collected using a revised standardized questionnaire based on the protocols of the National Health Survey (26). Duly trained nutrition students and nutritionists collected data. The participants underwent a peripheral vein puncture in the morning, after a 8-h fast, to assess the biochemical parameters, carried out by a trained nursing technician. 10 mL of blood were driven for biochemical (5 mL) and vitamin E status (5 mL) evaluation.

### 2.3 Anthropometric assessment

Weight, height, waist, hip circumference and blood pressure were measured. All measurements were performed on the same day, and the participants were barefoot, wearing light clothing and free of any adornments. For weight and height measurements, the volunteers were instructed to stand facing the assessor, with their heads aligned with the Frankfurt plane. BMI was calculated using the formula BMI = weight (Kg)/height (m)^2^. For BMI classification, the reference values proposed by the WHO were used (27). Waist circumference was measured with the individual in an orthostatic position and around the midpoint between the last rib and the iliac crest. Hip circumference was measured by circling the largest gluteal protuberance with the individual in the same position as mentioned above. Waist and hip circumferences were used to calculate the waist-to-hip ratio. The conicity index was determined using weight, height and waist circumference measurements, according to the mathematical equation proposed by Valdez (28). The visceral adiposity index was calculated with waist circumference, BMI, HDL-c, and triglycerides (29).

Body weight was measured using an electronic anthropometric scale (Líder^®^ model P200M) with a capacity of 150 kg and precision of 0.1 kg. Height was measured using a portable stadiometer (Avanutri^®^) with a range of 20–210 cm and a graduation of 0.1 cm, fixed to a base, with a stabilizer to lean against the wall. A 200 cm ergonomic tape measure (Cescorf^®^) was used to measure waist and hip circumference. Blood pressure was measured using an automatic upper arm blood pressure device (Comfort - HEM 7122 - BR2 Omron^®^ Healthcare, São Paulo, Brazil). The instruments were certified by the National Institute of Metrology, Standardization and Industrial Quality (INMETRO).

### 2.4 Evaluation of biochemical parameters

A total volume of 5 mL of blood was collected and stored in a tube without anticoagulant and with a separating gel, and then sent for analysis of total cholesterol and fractions, triglycerides, fasting glucose, fasting insulin and high-sensitivity C-reactive protein (hs-CRP), carried out by a reference laboratory.

Fasting blood glucose, total cholesterol and triglyceride levels were analyzed using enzymatic methods. High-density lipoprotein cholesterol (HDL-c) levels were measured using a homogeneous enzymatic colorimetric assay. Low-density lipoprotein cholesterol (LDL-c) values were calculated using the Friedewald formula [LDL-c = Total cholesterol - HDL-c + (Triglycerides/5)] (30). Non-HDL cholesterol (non-HDL-c) was determined using the difference between total cholesterol and HDL-c, as recommended by the Brazilian Society of Cardiology (31). Insulin was determined by sandwich immunoassay and hs-CRP by immunoturbidimetric assay. All these analyses were carried out using automated methods (COBAS 6000 - Roche^®^ Professional Diagnostics, Risch-Rotkreuz, Switzerland).

The homeostasis model assessment of insulin resistance (HOMA-IR) was calculated from the product of glycemia and insulinemia divided by a normalization factor [HOMA-IR = glucose (mg/dL) × 0.0555 × insulin (mUI/L)/22.5]. The cut-off of >2.71 was adopted, which is indicative of insulin resistance, according to Genoleze et al. (32). To calculate the Homeostasis Model Assessment of beta cell function (HOMA-B), the following equation was used: 20 × fasting insulin (μU/mL)/fasting glucose (mmol/L) − 3.5 (%) (33).

### 2.5 Determination of alpha-tocopherol serum concentrations

Blood was collected (5 mL) by peripheral venipuncture for the determination of serum alpha-tocopherol in tubes protected from light. The blood was collected in the morning after an 8-h fast by a trained nursing technician. The blood was then centrifuged at room temperature for 10 min (500 × *g*) to separate and remove the serum. After this, two aliquots from each participant were stored in an ultra-freezer at −80 °C until biochemical analysis. The serum was analyzed according to Ortega et al. (34), adapted by Ribeiro et al. (35). For 1.0 mL of serum, the same amount of 95% Ethanol (Vetec, Rio Daniele Ingrid Naihara França Priscila Gomes Eva Débora de Oliveira Andrade Oliveira Sousa Souza Marinho do Nascimento Janeiro, Brazil) and 2 mL of Hexane PA (Vetec, Rio de Janeiro, Brazil) were used. The samples were homogenized for 1 min in a vortex and centrifuged for 10 min at 4000 rpm to separate the supernatant layer, which was then removed to another tube. These steps of adding hexane and removing the supernatant were carried out three times to extract the alpha-tocopherol in the serum. Finally, 4 mL of the pooled layer of supernatant was evaporated in a water bath at 37 °C and the dry extract was dissolved in 250 μL of absolute ethanol (Vetec, Rio de Janeiro, Brazil) for analysis using High Performance Liquid Chromatography (HPLC). The concentrations of alpha-tocopherol in the samples were determined by HPLC on a Shimadzu chromatograph (Shimadzu, Kyoto, Japan), consisting of a 20 μL injector loop, CBM 20A communicator, LC-20 AT pump, SPD-20A UV-VIS detector, C18 LIChrospher^®^ 100 RP-18 column (5 μm) (Merck, Darmstadt, Germany) and a computer with LC Solution software (Shimadzu, Kyoto, Japan) for data processing. The chromatogram evolved in isocratic elution with a mobile phase of methanol in purity grade for HPLC and a flow rate of 1.0 mL per minute at a wavelength of 292 nm. The concentrations of alpha-tocopherol were identified and determined by comparison with the area of their respective standards (SigmaAldrich, São Paulo, Brazil), at a retention time of 10.3 min. The values in the samples were expressed in μmol/L and vitamin E deficiency was confirmed when serum alpha-tocopherol values below 12 μmol/L (<517 μg/dL) were found (36).

### 2.6 Usual dietary intake assessment

The usual dietary intake was estimated using two 24-h recalls (24hR). The 24hR were carried out on non-consecutive days, one face-to-face and the other by telephone survey, which made it possible to identify and quantify all the food, supplements and drinks consumed in the 24 h preceding the interview. The second 24hR was administered with an interval of 30–45 days between the application of the first recall, on different days of the week and at different times of the month, to avoid possible influences from the purchasing power of food and seasonality. For the present analysis, 79 participants presented two 24hR. Photographic records of home measurements and food portions were used during the individual interview to facilitate recording the amount of food consumed. This allowed the interviewees to point out which utensils were used in their daily lives and quantify the portions of food and drink consumed in small, medium, or large portions. A team of trained nutritionists collected the 24hR.

The preparations and their respective homemade measurements for each 24hR were organized in a Microsoft Excel^®^ spreadsheet (Microsoft Office, 2010) according to the meals eaten: breakfast, morning snack, lunch, afternoon snack, dinner and supper, as reported by the participants. The homemade measures of each preparation were converted into grams, milliliters and/or liters, according to the IBGE tables (37) and Pinheiro et al. (38) in that order. Recipes were standardized according to Maciel et al. (39). The standardized recipes contained the raw ingredients, cooking factors and the final amount of cooked ingredients, according to the weight of the cooked preparation eaten.

Then, foods were chemically analyzed using the Brazilian food composition tables: TBCA (2023) (40), TACO (2011) (41), IBGE (2011) (42) and Philippi (2002) (43), following that order. Nutritional information obtained directly from food labels was also considered when there was no information in the aforementioned tables and/or in the case of ultra-processed foods. The nutrients analyzed were: energy, fiber, protein, carbohydrates, total fat, saturated fat, monounsaturated fat, polyunsaturated fat and vitamin E.

Regarding the estimation of usual dietary intake, once the chemical analysis was completed, habitual consumption was assessed and intrapersonal variability was adjusted using the Multiple Source Method (MSM) (Department of Epidemiology, German Institute of Human Nutrition, Potsdam-Rehbrücke, version 1.0.1)^1^. Initially, the nutrients were tested for normality and those with a parametric distribution were energy, fiber, protein, carbohydrates, total fat, saturated fat, monounsaturated fat, polyunsaturated fat and vitamin E. These nutrients were then corrected for energy intake using the Statistical Package for the Social Sciences software (IBM SPSS Statistics 26). After adjusting for energy, the nutrients were tested again for normality, with fiber, protein, total fat, saturated fat, monounsaturated fat, polyunsaturated fat and vitamin E presenting non-parametric distribution and carbohydrates presenting parametric distribution.

### 2.7 Cardiovascular risk estimation

Cardiovascular risk was estimated using the Framingham global risk score (GRS), which assesses cardiovascular risk considering the following variables: gender, age, smoking, HDL-c, total cholesterol and systolic blood pressure. Individuals were classified according to the likelihood of having cardiovascular events in 10 years, as follows: (1) low risk: those with GRS < 5%; (2) intermediate risk: men with GRS between 5% and 20% and women with GRS between 5% and 10%; (3) high risk: men with GRS > 20% and women with GRS > 10% and (4) very high risk: individuals with atherosclerotic disease with or without clinical events (44).

### 2.8 Statistical analysis

The database was built using Microsoft Excel^®^ (Microsoft Office, 2010) and Statistical Package for Social Sciences version 23.0 (SPSS Inv. Chicago, IL). All quantitative variables were tested for normality using the Kolmogorov-Sminorv test. Variables with a normal distribution were represented as means (standard deviation) and those without a normal distribution were presented as medians (Q1; Q3). The Student’s *T*-test was used for variables with a normal distribution and the Mann-Whitney test for variables without a normal distribution to test whether there was a difference between the quantitative variables. The Chi-Square test was used to analyze categorical variables. The results were considered significant when they showed a significance level of less than 5% (*p* < 0.05).

Pearson’s coefficient (r) was used for variables with a normal distribution and Spearman’s coefficient (ρ) for variables without a normal distribution to determine the correlations between alpha-tocopherol serum concentrations and anthropometric, biochemical, dietary, and cardiovascular risk parameters variables. A multiple linear regression model was built considering alpha-tocopherol/total cholesterol serum concentrations as the dependent variable, considering the positive expected associations between alpha-tocopherol and total cholesterol (45–47). The independent variables in the final models were selected considering multicollinearity and those with *r* > 0.7 were excluded. Tolerance values above 0.10 and variance inflation factors below 10 were also considered when selecting the independent variables for the final model. Residual scatter plots were used to assess outliers, normality, linearity, homoscedasticity and independence of residuals. GRS, conicity index and usual total fat intake were the independent variables selected in the final model. The multiple linear regression model was presented with the β standardized regression coefficients, 95% confidence intervals (CI) and *p*-values. Power analysis was conducted *a posteriori* for the linear multiple regression, considering an effect size of 0.15, alpha at 0.05, number of predictors = 3, and total sample size = 92, using GPower software, and the achieved power was of 95.7%. The *post hoc* power analysis indicated that the sample size was sufficient to detect medium effect sizes with a power exceeding 80%, lending confidence to the statistical findings.

## 3 Results

Mean alpha-tocopherol serum concentrations in the study population was of 17.80 (8.43) μmol/L. The studied population was then stratified into two groups using alpha-tocopherol serum concentrations <P50 or ≥P50, and the socioeconomic, anthropometric, biochemical, dietary usual intake, and clinical characteristics of the 92 participants are shown accordingly in Table 1. There was a predominance of females in the studied population, but alpha-tocopherol serum concentrations were not associated with sex. Total cholesterol, non-HDL-c and LDL-c were significantly higher in individuals with higher serum alpha-tocopherol concentrations. The hs-CRP was significantly lower in individuals with higher concentrations of alpha-tocopherol. The GRS percentage was not different between the studied groups and was 10% for the total population. Usual dietary intake was also not different between the studied groups (Table 1).

**TABLE 1:** Socioeconomic, anthropometric, biochemical, clinical characteristics, and dietary usual intake of the study population according to alpha-tocopherol concentrations.

Variable	Total	Alpha-tocopherol concentrations (μ mol/L)		*p-value*
		<P50 n = 46	≥P50 n = 46	
**Socioeconomic variables**				
Income, median (Q1; Q3)	3000 (1470–7850)	3.000 (2.000–7.700)	2.750 (1.212–8.000)	0.647^b^
**Education, n (%)**				
None	4 (4.3)	2 (4.3)	2 (4.3)	0.552^c^
Elementary school	16 (17.4)	5 (10.9)	11 (23.9)	
High school incomplete	13 (14.1)	7 (15.2)	6 (13.0)	
High school complete	18 (19.6)	9 (19.6)	9 (19.6)	
Technical course	4 (4.3)	3 (6.5)	1 (2.2)	
Incomplete university degree	7 (7.6)	2 (4.3)	5 (10.9)	
Complete university degree	30 (32.7)	18 (39.2)	12 (26.1)	
**Gender, n (%)**				
Female	56 (60.9)	28 (60.9)	28 (60.9)	1.000^c^
Male	36 (39.1)	18 (39.1)	18 (39.1)	
**Physical exercise, n (%)**				
Yes	40 (43.5)	20 (43.5)	20 (43.5)	1.000^c^
No	52 (56.5)	26 (56.5)	26 (56.5)	
**Alcohol consumption, n (%)**				
Yes	36 (39.1)	19 (41.3)	17 (37.0)	0.669^c^
No	56 (60.9)	27 (58.7)	29 (63.0)	
**Smoking, n (%)**				
Yes	5 (5.4)	4 (8.7)	1 (2.2)	0.168^c^
No	87 (94.6)	42 (91.3)	45 (97.8)	
Age (years), median (Q1; Q3)	56 (38–67)	52 (37–66)	58 (40–67)	0.509^b^
**BMI, n (%)**				
Normal	38 (41.3)	18 (39.1)	20 (43.5)	0.524^c^
Overweight	21 (22.8)	9 (19.6)	12 (26.1)	
Obesity	33 (35.9)	19 (41.3)	14 (30.4)
**Anthropometric variables and blood pressure**				
Waist circumference (cm), mean (SD)	95.97 (14.76)	98.79 (15.26)	93.15 (13.83)	0.067^a^
Hip circumference (cm), median (Q1; Q3)	102.10 (96.10–111.30)	105.00 (97.50–113.00)	99.75 (95.00–108.60)	0.085^b^
Waist-to-hip ratio, mean (SD)	0.92 (0.08)	0.93 (0.07)	0.91 (0.09)	0.278^a^
Conicity index, median (Q1; Q3)	1.31 (1.25–1.36)	1.32 (1.26–1.37)	1.29 (1.20–1.36)	0.358^b^
Visceral adiposity index, median (Q1; Q3)	1.85 (1.10–3.05)	1.89 (1.25–2.85)	1.81 (1.04–4.15)	0.864^b^
Systolic blood pressure (mmHg), mean (SD)	128 (21)	126 (18)	130 (24)	0.386^a^
Diastolic pressure (mmHg), mean (SD)	79 (12)	78 (10)	80 (14)	0.480^a^
**Biochemical variables**				
Alpha-tocopherol (μmol/L), mean (SD)	17.80 (8.43)	11.20 (3.24)	24.40 (6.63)	<0.001^a^
Alpha-tocopherol/total cholesterol ratio (μmol/mmol), mean (SD)	3.48 (1.51)	2.40 (0.86)	4.57 (1.22)	<0.001^a^
Total cholesterol (mg/dL), mean (SD)	198 (39)	187 (39)	209 (37)	0.007^a^
TGL (mg/dL), median (Q1; Q3)	125 (90–176)	115 (92–153)	129 (89–215)	0.323^b^
HDL-c (mg/dL), median (Q1; Q3)	47 (39–61)	45 (39–55)	50 (40–64)	0.379^b^
Non-HDL-c (mg/dL), mean (SD)	147 (39)	138 (39)	157 (37)	0.021^a^
LDL-c (mg/dL), mean (SD)	120 (35)	112 (37)	127 (32)	0.038^a^
VLDL-c (mg/dL), median (Q1; Q3)	23.60 (18.00–33.80)	22.70 (18.40–29.00)	25.20 (17.80–39.80)	0.331^b^
HOMA-IR, median (Q1; Q3)	2.55 (1.38–4.54)	2.57 (1.38–5.14)	1.99 (1.38–4.08)	0.742^b^
HOMA-B, median (Q1; Q3)	129 (97–220)	142.20 (90.40–256.30)	127.30 (100.00–167.70)	0.268^b^
Fasting glucose (mg/dL), median (Q1; Q3)	90 (83–100)	90 (83–96)	92 (82–102)	0.623^b^
Fasting insulin (μU/mL), median (Q1; Q3)	11.00 (6.96–18.52)	11.01 (6.30–19.73)	9.91 (7.00–15.62)	0.679^b^
Hs-CRP (mg/dL), median (Q1; Q3)	0.18 (0.09–0.45)	0.21 (0.12–0.52)	0.12 (0.06–0.39)	0.038^b^
**Global risk score (GRS)**				
GRS percentage, median (Q1; Q3)	10.00 (3.30–17.15)	7.00 (2.80–15.90)	12.70 (3.90–18.50)	0.297^b^
Cardiovascular risk classification, n (%)				
Low risk	31 (33.7)	19 (41.3)	12 (26.1)	0.462^c^
Intermediate risk	29 (31.5)	12 (26.1)	17 (37.0)	
High risk	30 32.6)	14 (30.4)	16 (34.8)	
Very high risk	2 (2.2)	1 (2.2)	1 (2.2)	
**Self-reported chronic diseases**				
Depression, n (%)	21 (22.8)	7 (15.2)	14 (30.4)	0.082^c^
Diabetes, n (%)	15 (16.3)	8 (17.4)	7 (15.2)	0.778^c^
Allergy in general, n (%)	17 (18.5)	7 (15.2)	10 (21.7)	0.420^c^
Hypertension, n (%)	34 (37.0)	16 (34.8)	18 (39.1)	0.666^c^
**Usual dietary intake variables**	**Total**	**Alpha-tocopherol concentrations (μ mol/L)**		***p-value***
		**<P50** **n = 37**	**≥P50** **n = 42**	
Energy (kcal), median (Q1; Q3)	1781.93 (1455.12)	1752.75 (1470.69–1841.33)	1783.47 (1412.44–1820.92)	0.851
Carbohydrate (g), mean (SD)	224.14 (29.74)	227.21 (37.28)	221.44 (21.13)	0.393
Protein (g), median (Q1; Q3)	72.04 (69.52–79.52)	72.04 (68.86–80.42)	72.05 (68.97–76.54)	0.630
Fiber (g), median (Q1; Q3)	19.27 (17.56–19.82)	19.31 (16.50–19.82)	19.27 (17.99–19.74)	0.377
Total fat (g), median (Q1; Q3)	54.22 (51.68–58.09)	54.35 (49.94–58.63)	53.85 (52.62–57.15)	0.821
Saturated fat (g), mean (SD)	15.72 (3.78)	16.24 (4.46)	15.26 (3.04)	0.251
Monounsaturated fat (g), median (Q1; Q3)	14.51 (13.56–15.11)	14.51 (13.55–15.00)	14.51 (13.92–15.11)	0.768
Polyunsaturated fat (g), median (Q1; Q3)	2.78 (2.66–2.90)	2.78 (2.64–2.84)	2.79 (2.69–2.90)	0.666
Alpha-tocopherol (mg), median (Q1; Q3)	4.55 (4.38–5.49)	4.54 (4.37–5.16)	4.64 (4.39–5.76)	0.302

Legend: (SD), standard deviation; (Q1; Q3), (1st quartile; 3rd quartile); n, number of individuals in the sample; BMI, Body Mass Index; TGL, triglycerides; HDL, high density lipoprotein cholesterol; LDL, low-density lipoprotein cholesterol; VLDL, very low-density lipoprotein cholesterol; HOMA-IR, homeostasis assessment model of insulin resistance; HOMA-B, homeostasis assessment model of beta cell function; Hs-CRP, ultrasensitive C reactive protein.

^a^*T*-test;

^b^Mann-Whitney test;

^c^Chi-Square test. Values of *p* < 0.05 were considered statistically significant.

We further considered the alpha-tocopherol/total cholesterol ratio, and no significant correlations with anthropometric, biochemical, habitual dietary intake and cardiovascular risk variables were observed in the study population (Table 2).

**TABLE 2:** Pearson (r) and Spearman (ρ) correlations between alpha-tocopherol/total cholesterol ratio and anthropometric, biochemical, dietary usual intake, and cardiovascular risk variables in the studied population.

Anthropometric variables	Alpha-tocopherol/total cholesterol (μ mol/mmol)
BMI (kg/m^2^) (r)	−0.127
Waist circumference (cm) (r)	−0.164
Waist-to-hip ratio (r)	−0.128
Conicity index (ρ)	−0.134
Visceral adiposity index (ρ)	0.007
**Biochemical variables**	**Alpha-tocopherol/total cholesterol (μmol/mmol)**
TGL (mg/dL) (ρ)	0.002
HDL-c (mg/dL) (ρ)	−0.005
Non-HDL-c (mg/dL), (r)	−0.081
LDL-c (mg/dL) (r)	−0.114
VLDL-c (mg/dL) (ρ)	−0.014
HOMA-IR (ρ)	−0.027
HOMA-B (ρ)	−0.036
Fasting glucose (mg/dL) (ρ)	−0.002
Fasting insulin (μU/mL) (ρ)	0.002
HsCRP (mg/dL) (ρ)	−0.119
**Usual dietary intake variables**	**Alpha-tocopherol/total cholesterol (μmol/mmol)**
Energy (kcal) (ρ)	−0.048
Carbohydrate (g) (r)	−0.129
Protein (g) (ρ)	−0.100
Fiber (g) (ρ)	−0.107
Total fat (g) (ρ)	0.033
Saturated fat (g) (ρ)	−0.105
Monounsaturated fat (g) (ρ)	0.023
Polyunsaturated fat (g) (ρ)	−0.047
Alpha-tocopherol (mg) (ρ)	0.065
**Cardiovascular risk variable**	**Alpha-tocopherol/total cholesterol (μmol/mmol)**
% Global risk score (ρ)	0.009

BMI, Body Mass Index; TGL, triglycerides; HDL, high density lipoprotein cholesterol; non-HDL, non-high density lipoprotein cholesterol; LDL, low-density lipoprotein cholesterol; VLDL, very low-density lipoprotein cholesterol; HOMA-IR, homeostasis assessment model of insulin resistance; HOMA-B, homeostasis assessment model of beta cell function; Hs-CRP, ultrasensitive C-reactive protein. No significant correlations were found.

The multiple linear regression model showed that GRS was positively associated (β = 0.328; 95% CI 0.015, 0.100; *p* = 0.009) and the conicity index was negatively associated (β = −0.290; 95% CI −8.196, −0.728; *p* = 0.020) with the concentrations of the alpha-tocopherol/total cholesterol (Table 3).

**TABLE 3:** Multiple linear regression model between alpha-tocopherol/total cholesterol (μmol/L), global risk score (GRS), conicity index, and total fat.

Variable	Alpha-tocopherol/total cholesterol (μ mol/mmol)	
	Standardized β coefficients (95% CI)	*P*-value
Global risk score (%)	0.33 (0.02; 0.10)	0.009
Conicity index	−0.29 (−8.20; −0.73)	0.020
Usual total fat intake (g)	−0.01 (−0.05; 0.04)	0.903

Model summary: R^2^ = 0.108; F (3, 75) = 3.012, *p* = 0.035. *P*-values < 0.05 were considered statistically significant.

## 4 Discussion

The present study evaluated alpha-tocopherol serum concentrations and its relationship with anthropometric, biochemical, usual dietary intake, and cardiovascular risk parameters. As expected (45–47), alpha-tocopherol was positively associated with lipid fractions, such as total cholesterol, non-HDL-c and LDL-c. Participants with higher serum alpha-tocopherol exhibited significantly lower hs-CRP levels. In the multiple regression, GRS was positively associated and the conicity index negatively associated with alpha-tocopherol/total cholesterol concentrations. These results reinforce that alpha-tocopherol concentrations are associated with cholesterol and lipoproteins, adding to the existing literature that alpha-tocopherol serum concentrations may be correlated with inflammation and cardiovascular risk measured by the GRS.

As expected, our results showed that individuals with higher serum concentrations of alpha-tocopherol presented higher values of total cholesterol, non-HDL-c, and LDL-c. Vitamin E’s metabolism is associated with lipoproteins, and it is transported by lipophilic molecules that subsequently distribute alpha-tocopherol to the liver, organs, and tissues (45). Thus, the action of LDL-c can be highlighted as one of the main transporters of alpha-tocopherol, which may justify our findings (46).

Our findings showed that participants with higher serum alpha-tocopherol exhibited significantly lower hs-CRP concentrations. hs-CRP stands out as an important biomarker for detecting inflammation, infections and the risk of developing cardiovascular diseases (48). In this sense, the action of alpha-tocopherol may suppress the production of the inflammatory cytokine IL-6, which binds to receptors on liver cells and stimulates the synthesis of CRP (49). Another associated mechanism, which has not been fully elucidated, is the dephosphorylation of protein kinase C (PKC), since alpha-tocopherol can activate activation protein 1 (AP-1) which will dephosphorylate PKC and inhibit the proliferation of smooth muscle cells, reducing the production of reactive oxygen species by monocytes (50–52). Thus, alpha-tocopherol, due to its ability to modulate inflammatory and oxidative responses, acts by reducing pro-inflammatory signaling and, consequently, hepatic CRP synthesis (1, 49).

Regarding the relationship between vitamin E and cardiovascular risk parameters, the role of alpha-tocopherol in the prevention of CVD is widely discussed due to its antioxidant capacity and anti-inflammatory properties (21). In addition to their antioxidant action, tocopherols are also inhibitors of platelet aggregation and can act to inhibit thrombi (53). Observational studies have suggested that serum concentrations of alpha-tocopherol ≥ 30 μmol/L are associated with a possible protective effect against cardiovascular diseases (11). In the present study, the evaluated individuals presented a mean alpha-tocopherol serum concentration of 17.80 μmol/L. Considering that serum concentrations of α-tocopherol ≥ 30 μmol/L are indicative cardioprotective effect, the individuals evaluated in this study may be at increased risk of developing cardiovascular diseases.

Although we identified significant hs-CRP and other biochemical differences in the groups evaluated, no differences were observed in vitamin E usual dietary intake. This result can be attributed to factors that modify the bioavailability, metabolism and transport of this vitamin in the body. For example, the intestinal absorption of alpha-tocopherol is influenced by individual characteristics and the composition of the diet (54). Thus, the food matrix in which vitamin E is found represents an essential factor in its absorption. It is important to note that the amount of fat provided in the diet aims to facilitate the extraction of vitamin E from its food matrix, thereby stimulating bile secretion and promoting the formation of micelles (55). In the liver, the alpha-tocopherol transfer protein (alpha-TTP) directs alpha-tocopherol to plasma lipoproteins, allowing it to be distributed systemically (56). In addition, genetic polymorphisms, such as in the TTPA gene, which encodes alpha-TTP, can compromise the distribution of alpha-tocopherol, regardless of dietary intake (57).

In the present study, the linear regression showed that the percentage of GRS was positively associated with the alpha-tocopherol/total cholesterol serum concentrations. The GRS is a widely used tool for predicting the risk of cardiovascular events over a 10-years period and considers variables such as age, total cholesterol and its fractions, blood pressure, smoking and diabetes (44). The positive association found in our study can be explained by the fact that alpha-tocopherol circulates in the plasma by binding to lipoproteins, especially LDL and HDL – variables that are included in the GRS calculation. Therefore, individuals with dyslipidemia or hypercholesterolemia, conditions that increase the concentrations of these lipoproteins, may present higher serum concentrations of alpha-tocopherol. However, this increase may reflect greater action and availability of lipid transporters in the blood and not necessarily a protective effect of vitamin E on cardiovascular risk (58). In this sense, this association may reflect an increase in the transport of lipoprotein-bound alpha-tocopherol, particularly in individuals with high LDL cholesterol levels, rather than a causal cardioprotective effect. In addition, it is worth noting that individuals at higher cardiovascular risk may have greater oxidative stress and inflammation. In these conditions, there is a greater demand for vitamin E by the body due to its antioxidant action which protects cell membranes from lipid peroxidation and oxidative damage, which may result in an increase in serum vitamin E serum concentrations (59).

Some studies have found inverse associations between alpha-tocopherol serum concentrations and CVD mortality (60, 61). Huang et al. (23) followed men for a period of 30 years and observed that higher alpha-tocopherol serum concentrations correlated with a lower risk of general and specific mortality, including CVD causes. However, the literature also reports studies that did not observe this possible protective action of vitamin E, demonstrating that the comprehension of the relationship between vitamin E and CVD is still evolving (53, 62, 63). CVD diseases are multifactorial and complex, as is vitamin E metabolism, which is why more studies are needed to better elucidate these associations (7).

The multiple linear regression also showed that the conicity index was negatively associated with the alpha-tocopherol/total cholesterol ratio. The conicity index is a useful anthropometric indicator for assessing central adiposity and predicting the risk of cardiometabolic diseases using waist circumference, height, and weight measurements (28). The conicity index calculation implies that the accumulation of fat in the abdominal region alters body geometry, promoting the transition from a cylindrical shape to a shape like a double cone (64). When evaluating the associations between adiposity and serum concentrations of alpha-tocopherol, Szewczyk et al. (58) found that individuals with higher anthropometric measurements of waist circumference, hip circumference, and waist-to-hip ratio had lower alpha-tocopherol serum concentrations. Other studies have also reinforced this association between higher anthropometric measurements and lower alpha-tocopherol serum concentrations, which agrees with our findings (65, 66). These associations observed in individuals with obesity can be explained by different reasons, including increased oxidative stress, low dietary intake of foods that are sources of vitamin E, and the possible sequestration of alpha-tocopherol by adipose tissue (67, 68).

Limitations of this research include the cross-sectional nature of the study, which makes it impossible to establish cause-and-effect relationships. Therefore, the directionality of the associations cannot be assumed. For example, although an association between higher alpha-tocopherol and LDL serum concentrations was observed, it is not possible to infer whether high LDL levels lead to increased transport of alpha-tocopherol or whether alpha-tocopherol can influence LDL concentrations. Furthermore, it is important to emphasize that the linear regression model, despite revealing statistically significant associations, showed a modest effect size, reflecting that alfa-tocopherol should not be considered in isolation as a reliable predictor of cardiovascular risk. Other limitations of this study are the small sample size, which may have interfered with the identification of other statistically significant relationships, and convenience sampling, which may induce selection bias and compromise the representativeness of the sample. Women predominated among participants, and metabolic and hormonal characteristics differ between the sexes. However, alpha-tocopherol concentrations were not associated with sex in our analysis. Finally, although dietary intake and serum alpha-tocopherol were not correlated, it is important to note that all participants who used vitamin E supplements were excluded, minimizing the confusion caused by exogenous intake.

As strengths, few studies have investigated serum alpha-tocopherol concentrations and their relationship with anthropometric, biochemical, usual dietary intake, and cardiovascular risk parameters. In the literature, there are no studies that directly explored the associations between GRS and conicity index with alpha-tocopherol/total cholesterol serum concentrations. The available studies have observed associations between vitamin E and other variables related to cardiovascular disease and cardiovascular risk (69, 70). Thus, to date, this is the first study to find positive associations between GRS and alpha-tocopherol/total cholesterol serum concentrations, and negative associations between conicity index and alpha-tocopherol/total cholesterol. Moreover, recent data from Brazilian national surveys have shown percentages of individuals with overweight (approximately 60%) and obesity (approximately 25%), comparable to the sample in this study (71, 72). Therefore, our study’s results can contribute to the planning of strategies and public policies aimed at preventing and treating overweight/obesity and cardiovascular diseases, ultimately leading to lower costs for healthcare systems.

## 5 Conclusion

Our data demonstrated that serum alpha-tocopherol concentrations were positively associated with total cholesterol, non-HDL cholesterol, LDL cholesterol, and GRS, and were inversely related to hs-CRP levels. Additionally, the conicity index showed a negative association with the alpha-tocopherol/total cholesterol ratio. These findings suggest that alpha-tocopherol status is linked to both lipid profile and cardiovascular risk indicators in the study population. The results showed that the population studied may be more vulnerable to metabolic alterations associated with low-grade inflammation and greater cardiovascular risk. Further studies should assess the possible influence of other variables, such as age and hormone profile, on alpha-tocopherol serum concentrations and their relationship with overweight/obesity and cardiovascular risk parameters.

## Data Availability

The original contributions presented in this study are included in this article/supplementary material, further inquiries can be directed to the corresponding author.

## Footnotes

1
https://msm.dife.de/
